# Complete chloroplast genomes of 13 species of sect. *Tuberculata* Chang (*Camellia* L.): genomic features, comparative analysis, and phylogenetic relationships

**DOI:** 10.1186/s12864-024-09982-w

**Published:** 2024-01-24

**Authors:** Zhaohui Ran, Zhi Li, Xu Xiao, Mingtai An, Chao Yan

**Affiliations:** 1 College of Forestry, Guizhou University, Guiyang, China; 2 Bioaffiliationersity and Nature Conservation Research Center, Guizhou University, Guiyang, China

**Keywords:** Sect. *Tuberculata*, Cp genomes, Phylogenetic relationship, Features, Comparative analysis

## Abstract

**Background:**

Sect. *Tuberculata* belongs to *Camellia*, and its members are characterized by a wrinkled pericarp and united filaments. All the plants in this group, which are endemic to China, are highly valuable for exploring the evolution of *Camellia* and have great potential for use as an oil source. However, due to the complex and diverse phenotypes of these species and the difficulty of investigating them in the field, their complex evolutionary history and interspecific definitions have remained largely unelucidated.

**Results:**

Therefore, we newly sequenced and annotated 12 chloroplast (cp) genomes and retrieved the published cp genome of *Camellia anlungensis* Chang in sect. *Tuberculata*. In this study, comparative analysis of the cp genomes of the thirteen sect. *Tuberculata* species revealed a typical quadripartite structure characterized by a total sequence length ranging from 156,587 bp to 157,068 bp. The cp.genome arrangement is highly conserved and moderately differentiated. A total of 130 to 136 genes specific to the three types were identified by annotation, including protein-coding genes (coding sequences (CDSs)) (87–91), tRNA genes (35–37), and rRNA genes (8). The total observed frequency ranged from 23,045 (*C. lipingensis*) to 26,557 (*C. anlungensis*). IR region boundaries were analyzed to show that the *ycf1* gene of *C. anlungensis* is located in the IRb region, while the remaining species are present only in the IRa region. Sequence variation in the SSC region is greater than that in the IR region, and most protein-coding genes have high codon preferences. Comparative analyses revealed six hotspot regions (*tRNA-Thr(GGT)-psbD*, *psbE-petL*, *ycf15-tRNA-Leu(CAA)*, *ndhF-rpl32*, *ndhD*, and *trnL(CAA)-ycf15*) in the cp genomes that could serve as potential molecular markers. In addition, the results of phylogenetic tree construction based on the cp genomes showed that the thirteen sect. *Tuberculata* species formed a monophyletic group and were divided into two evolutionarily independent clades, confirming the independence of the section.

**Conclusions:**

In summary, we obtained the cp genomes of thirteen sect. *Tuberculata* plants and performed the first comparative analysis of this group. These results will help us better characterize the plants in this section, deepen our understanding of their genetic characteristics and phylogenetic relationships, and lay the theoretical foundation for their accurate classification, elucidation of their evolutionary changes, and rational development and utilization of this section in the future.

**Supplementary Information:**

The online version contains supplementary material available at 10.1186/s12864-024-09982-w.

## Background

*Camellia* is the largest genus of Theaceae; it consists primarily of evergreen shrubs or trees and contains more than 200 species in 18 sections according to the taxonomy of Chang et al. [1, 2]. Compared with other genera of Theaceae, *Camellia* is a relatively primitive lineage with many flowers and a basal phylogenetic position. *Camellia* species are distributed on both sides of the Tropic of Cancer in East Asia, with approximately 238 species present in China. Most of the species are found in Yunnan, Guangxi, Guangdong and Sichuan, China, and the rest are distributed in the Indo-China Peninsula and Japan, eastern India and the Philippines. *Camellia* species are typical representatives of the Chinese flora, and many *Camellia* species are endemic to China [1, 3]. These species have a high utilization value and are important sources of materials for tea and oil production [4]. For example, the leaves of *Camellia* species are used to make tea, which is one of the three most famous nonalcoholic drinks worldwide and is a vital commodity in global trade. In addition, some species of *Camellia* in China are essential woody oil plants. These fruits are often used to produce safe oil products with high economic value and abundant health benefits.

In sect. *Tuberculata* Chang belongs to *Camellia* and is named for the “tuberculiform protuberance on the surface of the ovary and fruit”; it is thus considered a specialized taxon within sect. *Tuberculata* that has retained its primitive shape [5]. In 1939, the famous botanist Chongshu Qian first discovered a *Camellia* plant with a raised capsule peel in Sichuan Province, China, and named it *Camellia tuberculata* Chien, after which it was merged into sect. *Pseudocamellia* Sealy Rev. [6]. It was not until 1981 that sect. *Tuberculata* Chang was first identified by Chang and is now considered to include six species [1]. Twelve new species were reported over the next decade [7]. The species were divided into two subgroups (subsect. *Tuberculata* Chang and subsect. *Nudicarpa* Chang) according to whether the ovary had a wrinkled pericarp [7]. Currently, the division of sects. *Tuberculata* into groups has been recognized by many *Camellia* taxonomists. However, there is notable taxonomic conflict regarding the interspecific taxonomic relationships of these plants. Min [5] eliminated the classification levels of “subgroup” and “lineage” in this group and merged the 18 species into 6 species and 4 varieties. Notably, these classifications are based only on morphological data and lack a basis on data from molecular biology and other disciplines. This problematic systematic classification of sect. *Tuberculata* has aroused great interest among researchers, who have started to search for various methods, including classical classification methods, to address this problem. At present, the widely accepted classification system for sects. *Tuberculata* [1, 3] is based on the morphological characteristics of the plants. However, traditional morphological identification of species can be affected by environmental and human interference, resulting in unreliable results. Therefore, the main reason for the divergence in the classification of sect. *Tuberculata* is the use of purely morphological traits [8], which greatly hinders the conservation and utilization of plant germplasm resources.

The chloroplast (cp) is an important energy converter that functions in higher plants and some algae to promote life activities and is rich in genetic information; furthermore, cp has evolved slowly in most angiosperms [9]. Because of their conserved overall structure and high base substitution rates, they are useful for resolving the kinship of plant species and clarifying the evolution of developmental systems [10, 11]. Although cp genes evolve slowly and are conserved in sequence and structure, narrowing and widening of their edges in IR regions are common and important factors contributing to cp genome length differences and structural variation. In contrast to the plant genome, the cp genome has a simple structure, a small molecular weight, multiple copies, conserved sequences, and slow evolution, as well as additional repetitive sequences and rich genetic information; these characteristics are useful for constructing a molecular phylogenetic tree [12, 13]. Furthermore, phylogenies constructed based on complete cp genome sequences are highly convincing and credible [14]. As next-generation DNA sequencing (NGS) technology has matured and sequencing prices have decreased, obtaining complete plasmid sequences in the laboratory has become common [15]. By searching the literature and the National Center for Biotechnology Information (NCBI) database, we found that only four complete cp.genomes in sect. *Tuberculata* have been reported [16–19]. This lack of data limits further exploration of this species.

In this study, we performed high-throughput sequencing on 12 samples of 12 species taken from the field. The cp genomes of sect. *Tuberculata* plants were analyzed in combination with information in the NCBI database. This study aimed to address the following: (1) perform a comparative evaluation of the genomes to discover structural aspects and variations in the sequences, (2) analyze codon preferences and cp genome diversity among species and identify hotspots with high codon usage preferences and high nucleotide diversity among species, and (3) construct a phylogenetic tree based on the cp genomes of the 13 sect. *Tuberculata* plants to preliminarily explore their phylogeny and relationships. In this way, we aimed to enrich the cp genome database of sect. *Tuberculata* and provide a basis for further taxonomic identification and utilization of sect. *Tuberculata* species.

## Results

### Chloroplast genome structure and features

**Fig. 1:**
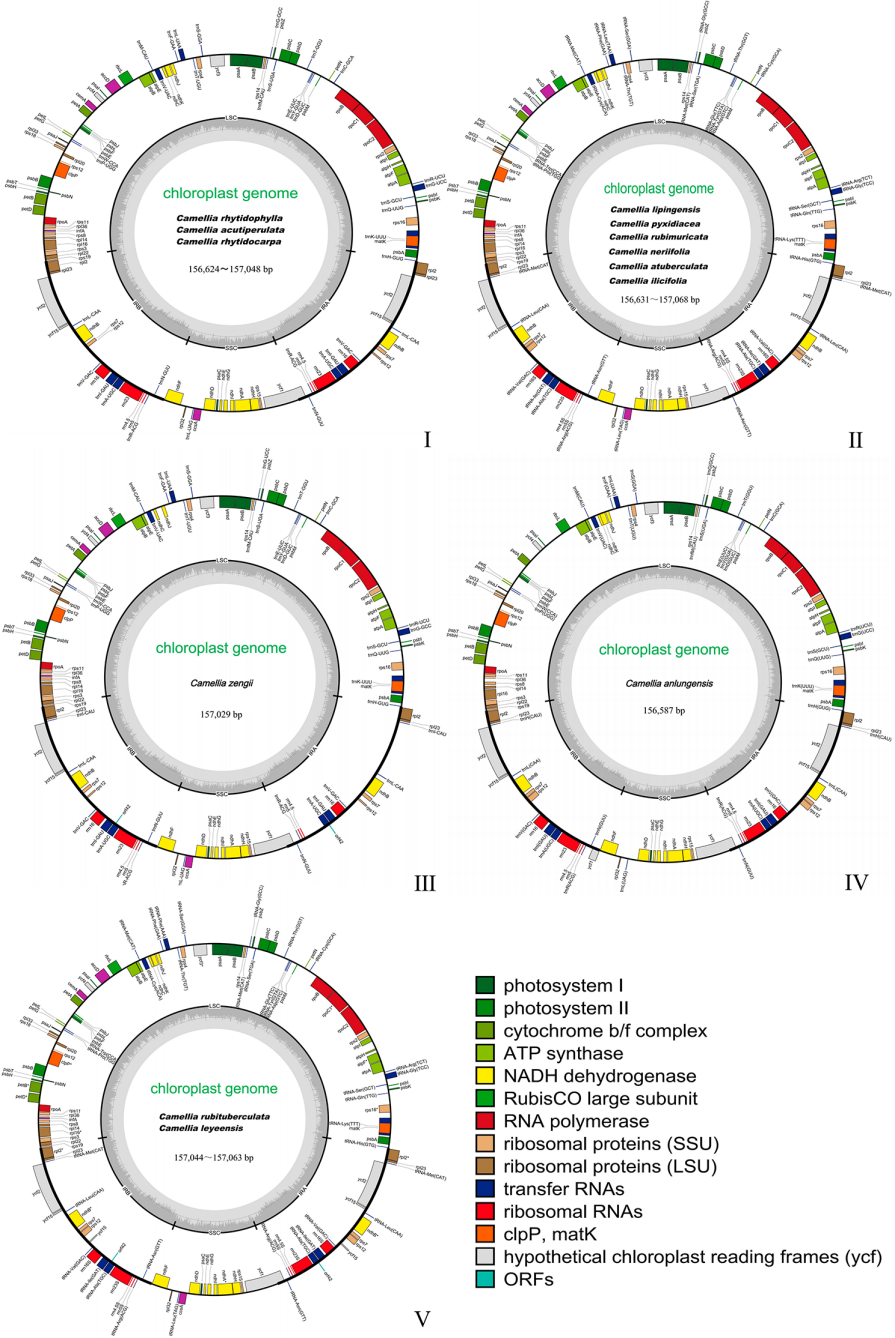
Maps of the thirteen sect. *Tuberculata* cp genomes

Comparative analysis of the cp genomes of the thirteen sect. *Tuberculata* species revealed a typical quadripartite structure characterized by a total sequence length of 156,587 bp (*C. anlungensis*) to 157,068 bp (*C. atuberculata*), including the LSC region (86,212−86,732 bp), the SSC region (18,276−18,339 bp), and two identical inverted repeat (IR) regions (51,986−52,130 bp) (Fig. 1; Table 1). Moreover, the thirteen sect. *Tuberculata* species were classified according to the number of genes, and five cp genome types were obtained (Fig. 1; Table 1). A total of 130 to 136 genes specific to the three types were obtained by annotation, including protein-coding genes (coding sequences (CDSs)), tRNA genes, and rRNA genes (87–91, 35–37, and 8, respectively). The GC content ranged from 37.30 to 37.34%. The SSC region (30.59–30.63%), LSC region (35.31–35.37%), and IR region (42.94–42.98%) are in order from small to large. The cumulative length of the CDSs ranged from 79,099 (*C. neriifolia*) to 80,175 bp (*C. leyeensis*), and the GC contents of the CDSs ranged from 37.52 to 37.65%. In addition, the first codon position, the second codon position, and the third codon position had the following order of GC content from low to high in the CDSs: third position (29.40-29.53%), second position (37.94-38.04%), and first position (45.19-45.42%) (Table 1).

**Table 1:** Structure of the complete cp genomes of sect. *Tuberculata* plants

	*C.* *rhytidophylla*	*C.* *acutiperulata*	*C.* *rhytidocarpa*	*C.* *lipingensis*	*C.* *pyxidiacea*	*C.* *rubimuricata*	*C.* *neriifolia*	*C.* *atuberculata*		*C.* *ilicifolia*	*C.* *zengii*	*C.* *anlungensis*	*C.* *rubituberculata*	*C.* *leyeensis*
Genome size (bp)	156,625	156,624	157,048	157,011	156,677	156,631	157,067	157,068		157,067	157,029	156,587	157,044	157,063
GC (%)	37.34	37.33	37.31	37.30	37.33	37.33	37.30	37.31		37.31	37.31	37.33	37.31	37.30
LSC size (bp)	86,227	86,212	86,648	86,732	86,261	86,237	86,675	86,675		86,674	86,630	86,262	86,689	86,661
SSC size (bp)	18,282	18,282	18,282	18,281	18,286	18,276	18,282	18,283		18,283	18,281	18,339	18,279	18,284
IR size (bp)	52,116	52,130	52,118	51,998	52,130	52,118	52,110	52,110		52,110	52,118	51,986	52,076	52,118
GC of LSC (%)	35.37	35.36	35.33	35.32	35.35	35.37	35.31	35.31		35.31	35.33	35.31	35.31	35.31
GC of SSC (%)	30.60	30.59	30.62	30.63	30.62	30.59	30.61	30.61		30.61	30.62	30.60	30.62	30.60
GC of IR (%)	42.95	42.95	42.95	42.96	42.95	42.94	42.96	42.97		42.97	42.95	42.96	42.98	42.96
GC of CDSs (%)	37.55	37.61	37.54	37.53	37.53	37.52	37.61	37.61		37.61	37.61	37.54	37.65	37.55
1st position GC (%)	45.26	45.37	45.23	45.23	45.19	45.34	45.37	45.37		45.37	45.34	45.24	45.42	45.26
2nd position GC (%)	37.97	38.04	37.98	37.94	37.94	37.94	38.04	38.03		38.04	38.04	37.97	38.00	37.97
3rd position GC (%)	29.40	29.43	29.40	29.42	29.43	29.43	29.46	29.43		29.43	29.46	29.40	29.53	29.40
Length of CDSs	79,487	79,500	79,659	79,753	79,767	79,925	79,099	79,161		79,160	79,395	79,671	80,155	80,175
Number of genes	130	130	130	132	132	132	132	132	132		133	134	136	136
Number of CDSs	87	87	87	87	87	87	87	87	87		88	89	91	91
Number of tRNAs	35	35	35	37	37	37	37	37	37		37	37	37	37
Number of rRNAs	8	8	8	8	8	8	8	8	8		8	8	8	8
Genome type	I	I	I	II	II	II	II	II	II		III	IV	In	In

### Repeat structure and simple sequence repeat analyses

**Fig. 2:**
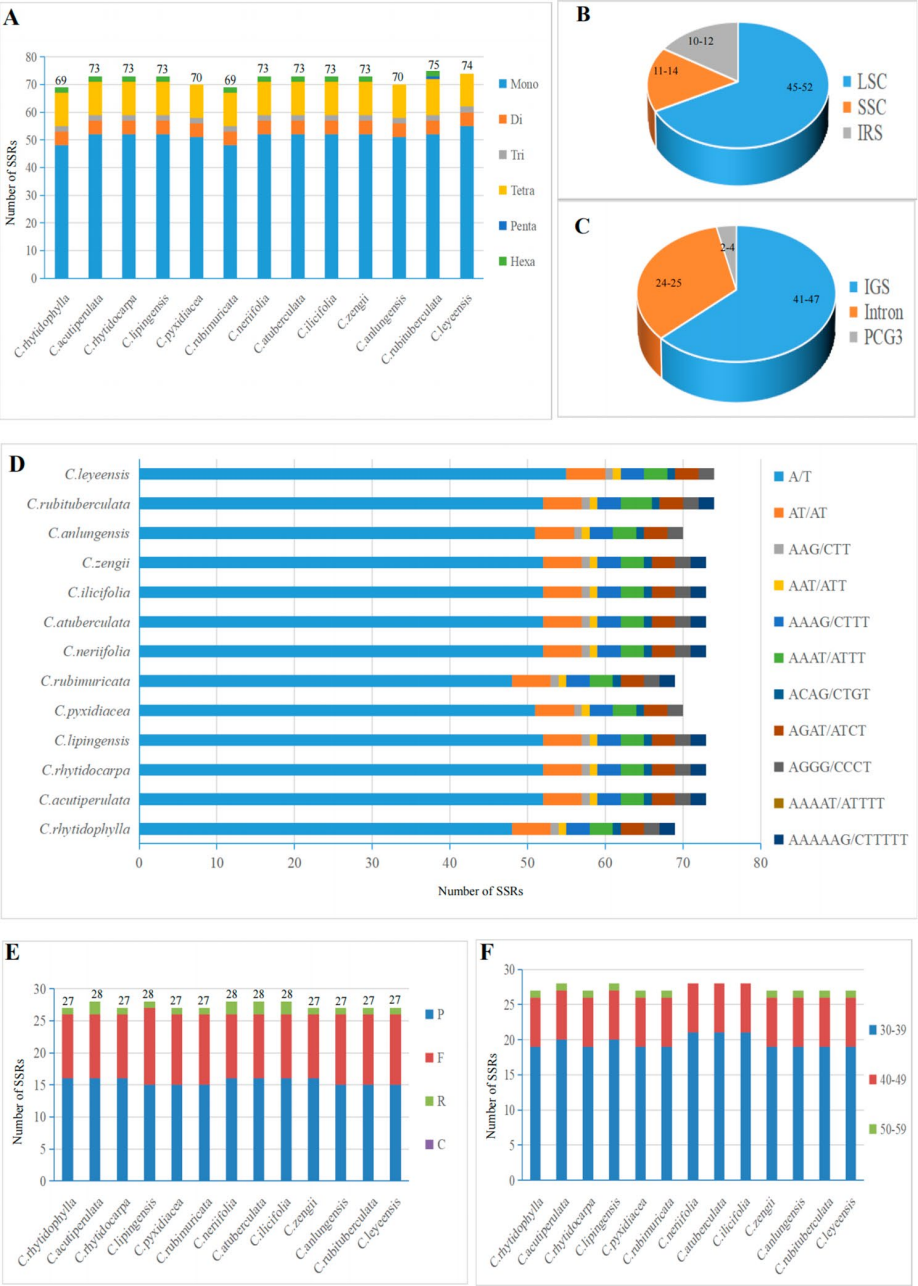
Comparative analysis of cp genome repeat sequences of thirteen species from sect. *Tuberculata*. **(A)**: The numbers of the six SSR types; (**B)**: The numbers of SSRs distributed in different copy regions; **(C)**: The numbers of SSRs distributed in different gene regions; **(D)**: The numbers of different SSR repeat unit types; **(E)**: The numbers of the four long repeat types. **(F)**: The length of the four long repeat types

In this study, a total of 69 (*C. rhytidophylla* and *C. rubimuricata*) to 75 (*C. rubituberculata*) SSRs of six types were detected, including 48–55 mononucleotides (mono-), 5 dinucleotides (di-), 2 trinucleotides (tri-), 12 tetranucleotides (tetra-), 0–1 pentanucleotide (penta-), and 0–2 hexanucleotides (hex-) (Fig. 2A). Among them, one pentanucleotide distributed in the IR region, AAAAT/ATTTT, was found only in *C. rubituberculata* (Fig. 2D). A statistical evaluation of all the identified SSRs revealed 45–52, 11–14, and 10–12 SSRs in the LSC, SSC, and IR regions, respectively (Fig. 2B). In addition, we found large differences in the distribution of these SSRs, with the largest number of SSRs occurring in intergenic sequences (IGSs) (41–47), followed by CDSs (24–25) and introns (2–4) (Fig. 2C). Among the six SSR repeat types, A/T was the only single-nucleotide SSR type, and the remaining five types of repeat units consisted mainly of A or T (Fig. 2D). These long repeat sequences ranged from 30 to 50 bp in length (Fig. 2F). Analysis of the scattered repeats with REPuter identified 27–28 scattered repeats, and no complementary (C) repeats were found. (Fig. 2E).

### Contraction and expansion of IR boundaries

**Fig. 3:**
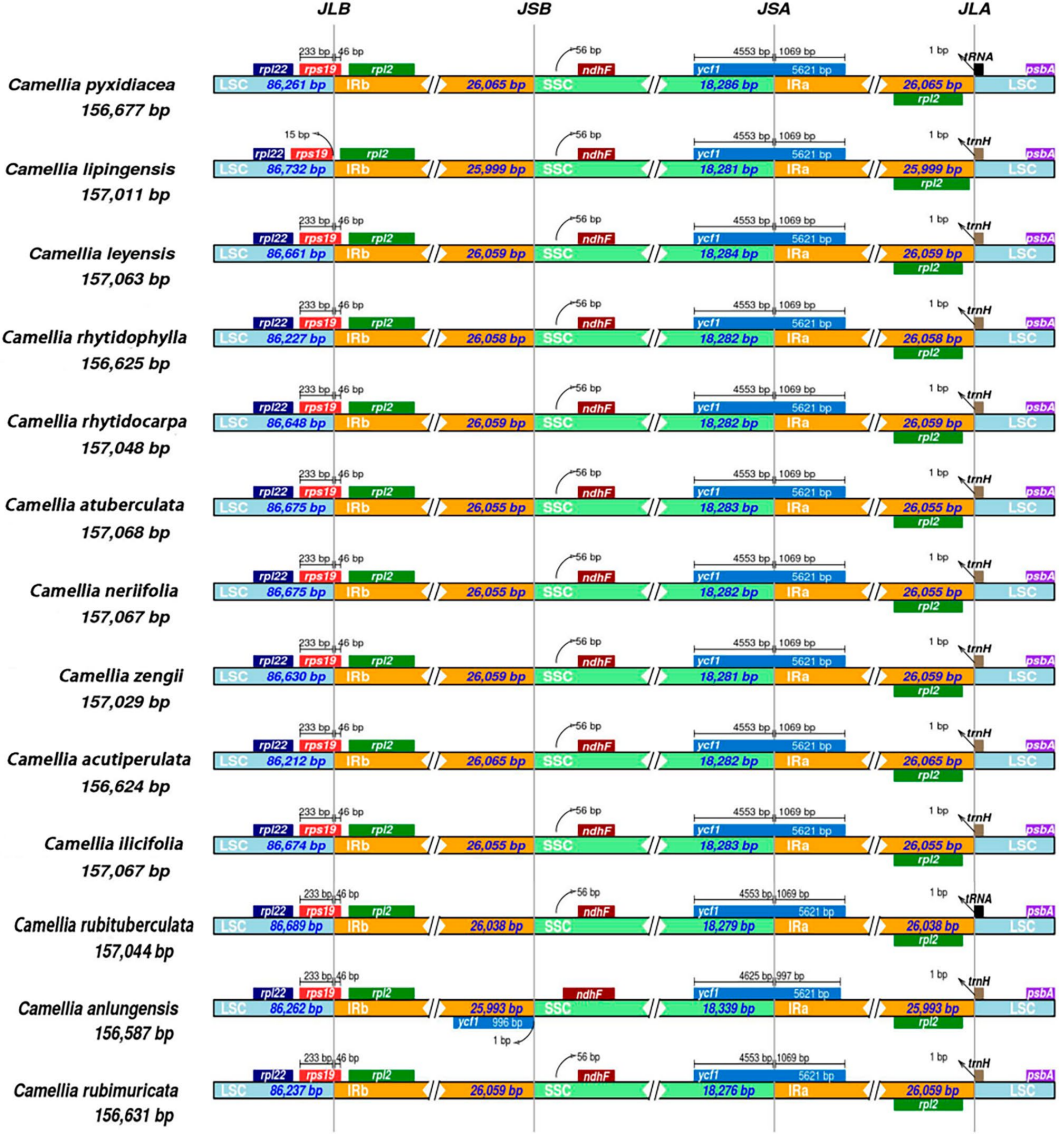
Analysis of the cp genome IR region boundaries of the thirteen species of sect. *Tuberculata*

Narrowing and widening of the edge of the IR region are very common; this phenomenon has become a popular topic of research and is the major driver of size differences between cp genomes [20, 21]. To clarify the spreading status of the IR region boundaries, the boundaries of the cp genomes of the thirteen sect. *Tuberculata* species were compared in this study. The results showed that the cp genome was conserved. However, there were still some structural variations in the various boundary zones (Fig. 3). In the sect. *Tuberculata* plants, the LSC/IRb boundary was located within the *rps19* gene, 46 bp of which extended into the IRb region. However, the *rps19* gene of *C. lipingensis* did not extend into the IRb region, and this region was separated from the LSC/IRb border by 15 bp.

The SSC/IRa boundary of *C. rubituberculata*, *C. rubimuricata*, *C. anlungensis*, *C. pyxidiacea*, *C. lipingensis*, *C. neriifolia*, *C. rhytidophylla*, *C. rhytidocarpa*, *C. atuberculata*, *C. acutiperulata*, *C. ilicifolia*, *C. zengii* and *C. leyeensis* was located in *ycf1*. In *C. anlungensis*, 997 bp of *ycf1* extended into the IRa region, while in the other species, the extension length was 1,069 bp. Interestingly, in *C. anlungensis*, 996 bp of the *ycf1* gene was located in the IRb region 1 bp from the SSC region. All the *rps19* genes were located at the LSC/IRb boundary, with *rps19* from *C. lipingensis* differing by 15 bp from the IRb region and all the other species extending 46 bp toward the IRb region. The *ndhF*, *tRNA* and *trnH* genes located in the SSC and LSC regions in the thirteen cp genomes showed no expansion to the IR regions. The IR regions of *C. rubituberculata* and *C. pyxidiacea* were located in the *tRNA* gene coding area, and those of *C. rubimuricata*, *C. lipingensis*, *C. leyeensis*, *C. anlungensis*, *C. neriifolia*, *C. rhytidophylla*, *C. rhytidocarpa*, *C. atuberculata*, *C. acutiperulata*, *C. ilicifolia* and *C. zengii* were located in the *trnH* gene (Fig. 3).

### Comparative cp Genome analysis

**Fig. 4:**
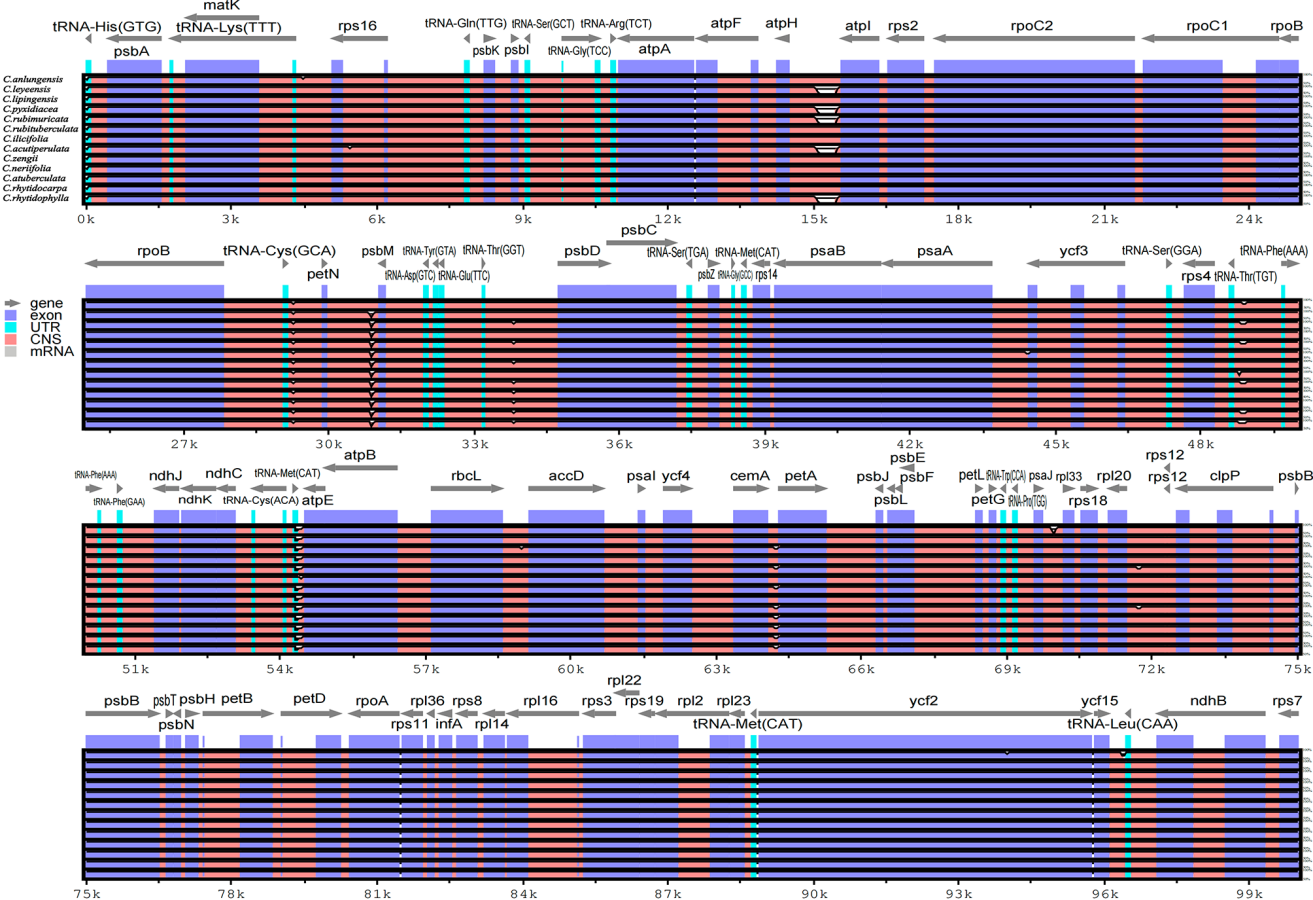
Sequence identity plot of the cp genomes of the thirteen sect. *Tuberculata* species. The *C. szechuanensis* sequence was used as a reference

In this study, the cp genomes of the thirteen sect. *Tuberculata* species were visually compared with those of mVISTA, and the *C. szechuanensis* was selected as a reference. The results showed that the cp genomes of the thirteen sect. *Tuberculata* species were conserved. Moreover, there was a difference in the IR region compared with the LSC and SSC regions, and the same was true for the noncoding and coding regions (Fig. 4). The regions with greater variation were located mainly in *tRNA-His(GTG)-psbA*, *atpH-atpl*, *petN-psbM*, *tRNA-Thr(TGT)-tRNA-Phe(AAA)*, and *tRNA-Met(CAT)-atpE*.

**Fig. 5:**
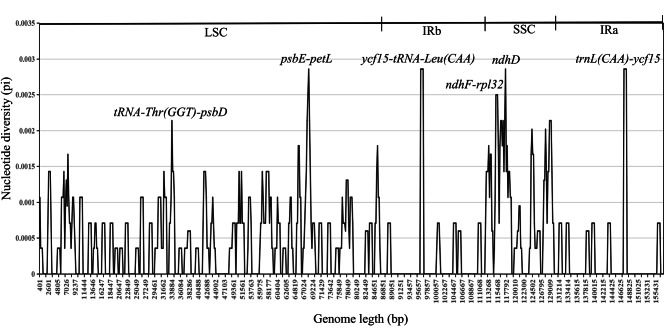
Comparison of nucleotide diversity (Pi) values among the thirteen sect. *Tuberculata* species in each section (window length: 800 bp; step size: 200 bp)

DNA molecular markers are usually highly variable regions of sequences that can be used for the differentiation of relationships between species. Therefore, to further understand the DNA polymorphisms (Pi), mutation hotspot regions in the cp genomes of the thirteen sect. *Tuberculata* plants were screened using DnaSP (Fig. 5). Pi analysis revealed that the pi values ranged from 0 to 0.00286, and the cp genome was relatively structurally conserved, small, and highly variable among the species. A total of six mutation hotspot regions (Pi > 0.0022) were detected, and they can be used as potential molecular markers. Among these, *tRNA-Thr(GGT)-psbD* and *psbE-petL* were located in the LSC region; *ndhF-rpl32* and *ndhD* were located in the SSC region; and *trnL (CAA)-ycf15* and *ycf15-tRNA-Leu (CAA)* were located in the IR region (Fig. 5).

### Codon usage

**Fig. 6:**
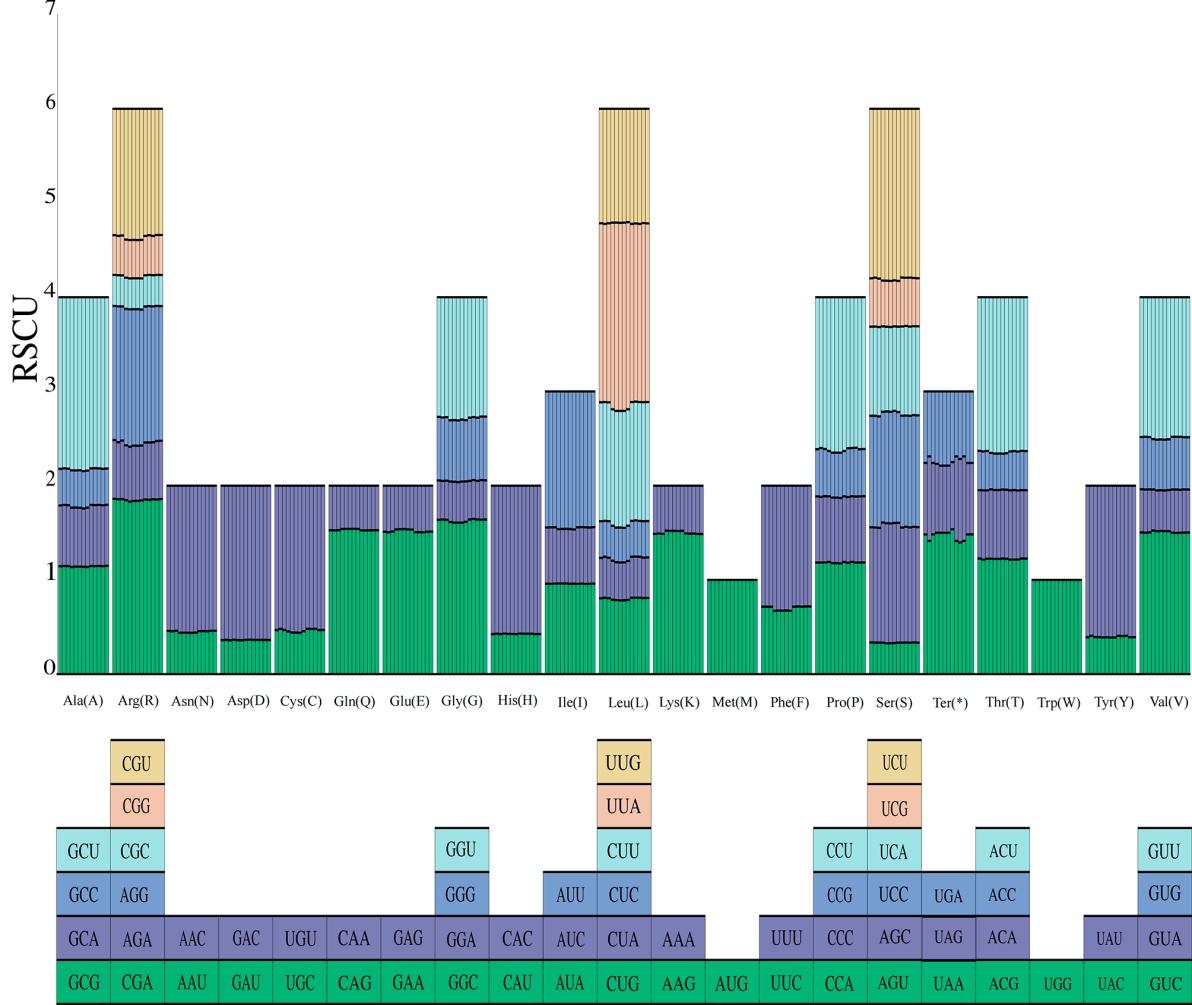
Codon content for 20 amino acids and stop codons in all the protein-coding genes of the cp genomes of the thirteen sect. *Tuberculata* species

Codon usage preferences are developed by organisms during long-term evolution and reflect the combined effects of genetics, evolution, and mutation on genes and species [22, 23]. In this study, the Thirteen sect. *Tuberculata* cp genomes were statistically analyzed and visually mapped based on relative synonymous codon usage (RSCU) (Fig. 6). A total of 64 different RSCU values were obtained for each species. For each of the thirteen sect. *Tuberculata* species, 64 codons were detected, and the remaining 61 codons edited 20 amino acids, except for the stop codons UAA, UAG, and UGA. The total observed frequency ranged from 23,045 (*C. lipingensis*) to 26,557 (*C. anlungensis*) (Table S2). Notably, both methionine and tryptophan have only one synonymous codon, while leucine, serine, and arginine have six synonymous codons.

In sect. *Tuberculata*, the most common encoded amino acid is leucine, the least common encoded amino acid is cysteine, the most common codon is UUU, and the least common codon is GCG. A codon with an RSCU value greater than 1.00 was the preferred codon. In addition, many preferred codons end in A or T, while nonpreferred codons end in C or G, which suggests a reduction in GC content in the coding region, which is a widespread phenomenon [24, 25].

### Phylogenetic analysis

**Fig. 7:**
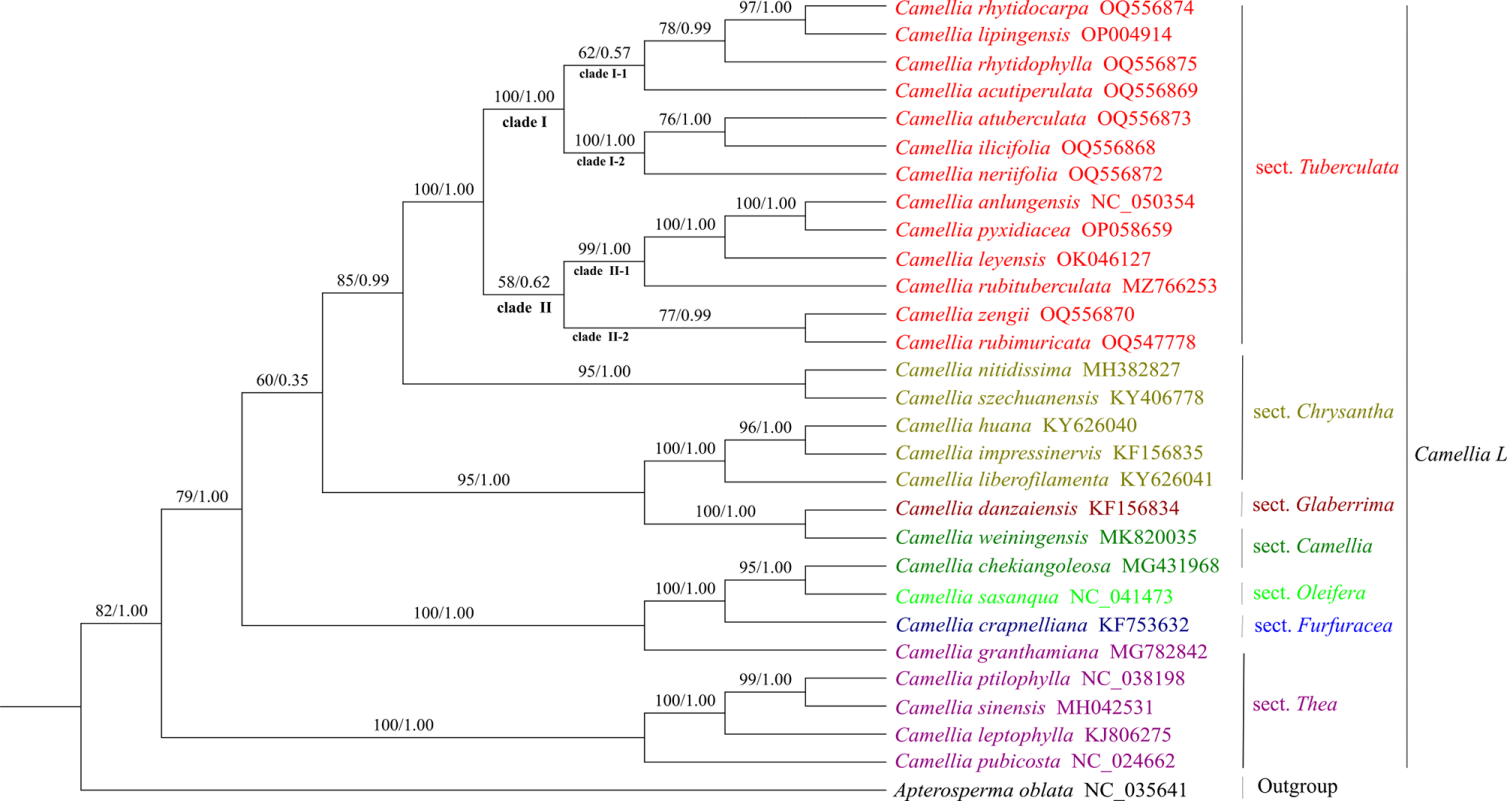
Phylogenetic tree obtained using the maximum likelihood (ML) and Bayesian inference (BI) methods for the sect. *Tuberculata* species based on complete cp genomes

A phylogenetic tree was constructed using 29 cp genomes, and the phylogenetic trees constructed by the ML method and the BI method were merged, illustrating the genetic relationships of the species (Fig. 7). The thirteen species from sect. *Tuberculata* used in this study, another 15 species and 1 outgroup are shown. In the phylogenetic trees, all plants in section *Tuberculata* were clustered on one large branch, and the plants in section *Tuberculata* were divided into groups different from those in other groups of *Camellia* L., confirming the independence of this section. In addition, the thirteen species of plants belonging to sect. *Tuberculata* were divided among two typical subbranches and four minor branches. Sect. *Tuberculata* was divided into four small branches: (clade I-1) *C. rhytidophylla*, *C. rhytidocarpa*, *C. atuberculata* and *C. lipingensis* on a branch (BS, PP = 62%, 0.57, respectively); (clade I-2) *C. atuberculata*, *C. ilicifolia* and *C. neriifolia* on a branch (BS, PP = 100%, 1.00, respectively); (clade II-1) *C. rubituberculata*, *C. anlungensis*, *C. pyxidiacea* and *C. leyeensis* on a branch (BS, PP = 99%, 1.00, respectively); and (clade II-2) *C. rubimuricata* and *C. zengii* on a branch (BS, PP = 77%, 0.99, respectively).

**Fig. 8:**
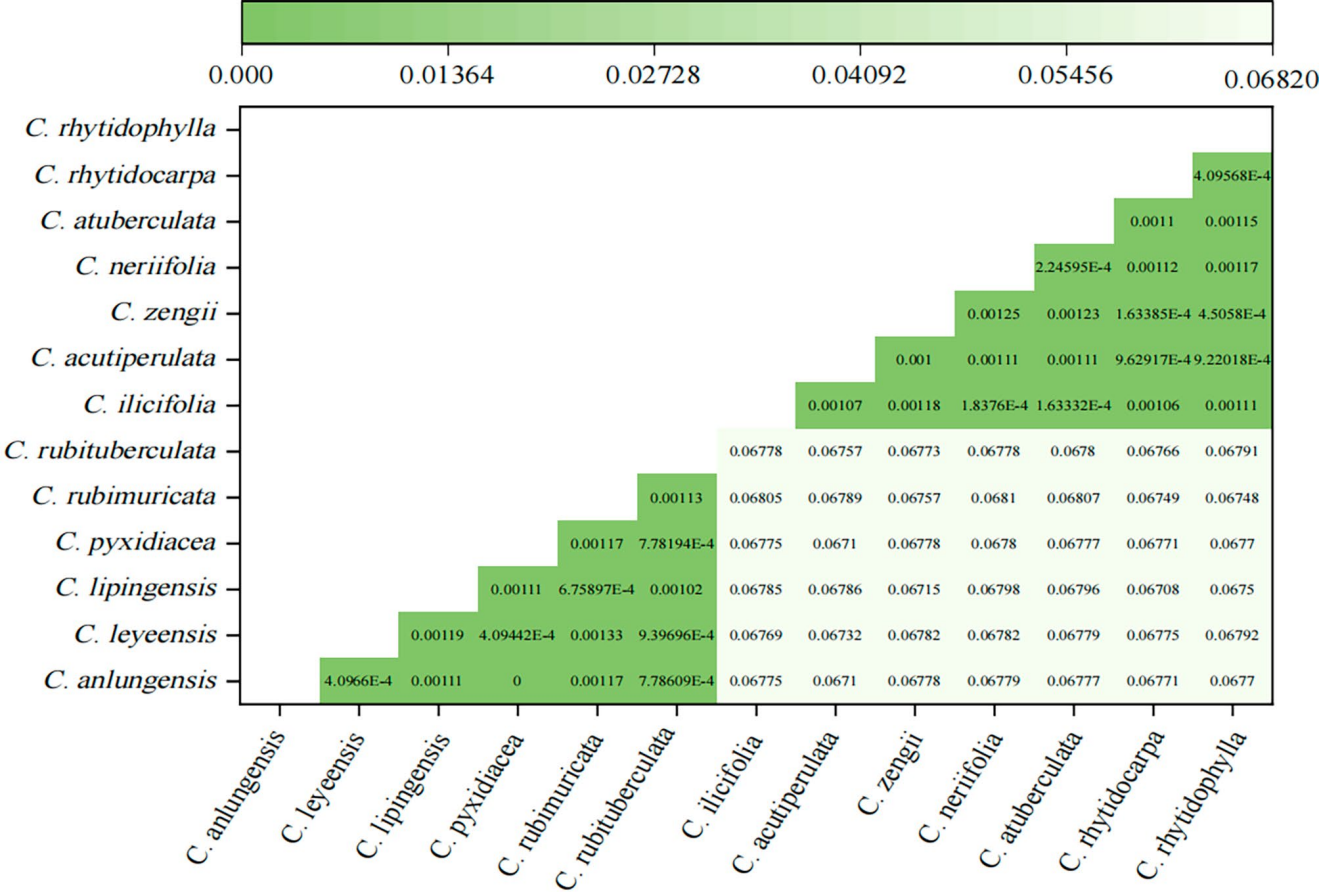
Genetic distances of the cp genomes of sect. *Tuberculata*

Among these species, *C. anlungensis* and *C. pyxidiacea* formed a subgroup distinct from *C. leyeensis*, which further supports the idea that they come from the same origin and are evolutionarily related. Additionally, *C. leyeensis* and *C. rubituberculata* converged on the same small branch. *C. rhytidocarpa* first matched *C. lipingensis* and then gradually joined *C. rhytidophylla* and *C. atuberculata*. The reliability of the phylogenetic tree was further validated by the genetic distance of the sect. *Tuberculata* cp genomes, which ranged from 0.00000 (*C. anlungensis* and *C. pyxidiacea*) to 0.06805 (*C. rubimuricata* and *C. ilicifolia*) (Fig. 8).

## Discussion

Previous studies have shown that the length of land plant cp genomes ranges from 120 to 170 kb [26]. In this study, comparative analysis of the cp genomes of the thirteen sect. *Tuberculata* species revealed a typical quadripartite structure characterized by a total sequence length of 156,587 bp (*C. anlungensis*) to 157,068 bp (*C. atuberculata*), including the LSC region (86,212−86,732 bp), the SSC region (18,276−18,339 bp), and two identical inverted repeat (IR) regions (51,986−52,130 bp). The GC content of the thirteen sect. *Tuberculata* plants ranged from 37.30 to 37.34%. In particular, in agreement with the results of many studies on angiosperms, the GC content was highest in the IR regions [27]. The differences in the cp genome among the species were obvious and attributed to changes in base composition. The conversion between sequences and higher GC content may be important factors leading to greater conservation of IR regions [28, 29].

For cp genomes, long repeat sequences facilitate the integration of transferred plasmid sequences into the host genome [30]. In this study, we found 69 (*C. rhytidophylla* and *C. rubimuricata*) to 75 (*C. rubituberculata*) SSR loci, and the LSC regions accounted for approximately 68% of the loci. The mononucleotide A/T was the most common, with a frequency of 71.64–74.32% in the cp genomes of sect. *Tuberculata*, consistent with values in other genera [31, 32]. Additionally, the cp SSRs in sect. *Tuberculata* also exhibited a high A/T content, which was positively correlated with the variability in cp genome sequences [33]. In conclusion, the statistical analysis of SSRs and interspersed repeats in the 13-sect. *Tuberculata* cp genomes laid the foundation for identification of similar molecular markers, genetic modifications, and species in sect. *Tuberculata*.

In angiosperms, the IR region is relatively conserved in sequence and structure, and the narrowing and widening of its edges are not only important factors for length variation but also the main cause of the emergence of pseudogenes [34, 35]. Although cp genes evolve slowly and are relatively conserved in terms of sequence and structure, boundary contraction and expansion in the IR regions are common phenomena. In this study, the LSC/IRb boundaries were located within the *rps19* gene, 46 bp of which extended into the IRb region. However, the *rps19* gene did not extend into the IRb region in *C. lipingensis*, and the region was separated from the LSC/IRb boundary by 15 bp. Another interesting finding was that *ycf1* was distributed at different boundaries. Therefore, the *ycf1* gene influences the expansion or contraction of the IR boundary in sect. *Tuberculata*.

For all the studied species in sect. *Tuberculata*, the 64 PCGs encoded 223,045 to 26,557 codons. These results were comparable to those in *Trapa*, in which 85 genes were found to encode 26,160−26,590 codons [36]. All thirteen species exhibited a highly conserved cp genome in sect. *Tuberculata*, as in *Trapa* [36], where the RSCU value of a single amino acid increased with the number of codons encoding that amino acid. Furthermore, the preference for half of all codons ending in A/T may be related to the high A/T content in the cp genome [37].

Molecular markers have many distinct advantages over conventionally applied genetic markers. By selecting highly polymorphic loci for DNA molecular markers, effective support for species kinship identification and gene pool construction can be provided [38, 39]. A good DNA barcode must be a short fragment of DNA that is representative of the species and is highly variable and amenable to amplification [40]. In our study, both the sequence and structure of the sect. *Tuberculata* cp genomes were highly conserved. mVISTA revealed that most of the species in sect. *Tuberculata* were structurally similar, except for *C. leyeensis*, *C. pyxidiacea*, *C. rubimuricata*, *C. acutiperulata*, and *C. rhytidophylla*. We observed that most of the variation in nucleotide sequences occurred in noncoding regions, which is the same as the findings of previous reports, from which we can infer that this variation may be a basic feature of angiosperms [41–43]. In addition, based on nucleotide diversity (Pi) analysis, six regions with large hotspot areas were identified as key loci for genetic studies, including five intergenic regions (*tRNA-Thr(GGT)-psbD*, *psbE-petL*, *ycf15-tRNA-Leu(CAA)*, *ndhF-rpl32*, and *trnL(CAA)-ycf15*) and one genic region (*ndhD*). In conclusion, these mutation hotspot regions will play an important role in the identification and characterization of plant species in sect. *Tuberculata*.

The cp genome is the main object of molecular biology research and has become a current research hotspot for species genealogy identification. In particular, phylogenetic analysis via whole-genome sequencing has become an important tool due to the improvements in sequencing technology and low cost. Phylogenetic trees constructed based on a single or a few gene sequences can have inconsistent or even conflicting topologies due to differences in evolutionary rates and horizontal shifts between genes, making it difficult to fully determine the correct evolutionary relationships of species [44, 45]. In this study, a phylogenetic tree was constructed using the BI method and the ML method. The cp genomes of the thirteen sect. *Tuberculata* species converged into a branch with high support. *C. rhytidocarpa* was similar to *C. lipingensis* and may be the same species. *C. ilicifolia* was classified on the same branch as *C. atuberculata* and *C. neriifolia* (BS and PP = 100% and 1.00, respectively), indicating that the three are similar and supporting the merger of *C. ilicifolia* into sect. *Tuberculata* [3]. The classification of *C. atuberculata*, *C. neriifolia* and *C. ilicifolia* as different species was supported [1]. *C. acutiperulata*, *C. anlungensis*, *C. pyxidiacea*, *C. leyeensis*, *C. rubimuricata*, *C. zengii* and *C. rubituberculata* formed a monophyletic group, which suggested that *C. pyxidiacea* and *C. rubituberculata* are separate species and that *C. anlungensis* and *C. leyeensis* are separate species [3]. On the basis of the phylogenetic tree and genetic distance results, we speculate that *C. anlungensis*, *C. pyxidiacea*, and *C. leyeensis* evolved from *C. rubituberculata.* Together, *C. zengii* and *C. rubimuricata* form a small branch, but the support rate is low, with support for only the independent taxonomic statuses of the two species [1]. As shown in our study, high-resolution cp genome sequences provide resources for broad research on the genetic information and species identification of sect. *Tuberculata*.

## Conclusion

In this study, for the first time, the whole cp genomes of thirteen species of plants in sect. *Tuberculata* were sequenced and compared. These cp genomes all had relatively conserved quadripartite structures. The repeat sequences, codon usage, and mutation hotspot regions of sect. *Tuberculata* were compared and analyzed, and six mutation hotspot regions were found to be potential molecular markers in this section. Phylogenetic trees and genetic distances were initially used to explore the affinities within sect. *Tuberculata*. The independent taxonomic status of the section was highly supported, with the thirteen species divided into two typical subclades and four minor clades. In summary, these results not only fill the data gap regarding the cp genome of sect. *Tuberculata* but also provide an important basis for the comprehensive exploration of cp phylogenetic relationships and the resolution of taxonomic and identification problems.

**Fig. 9:**
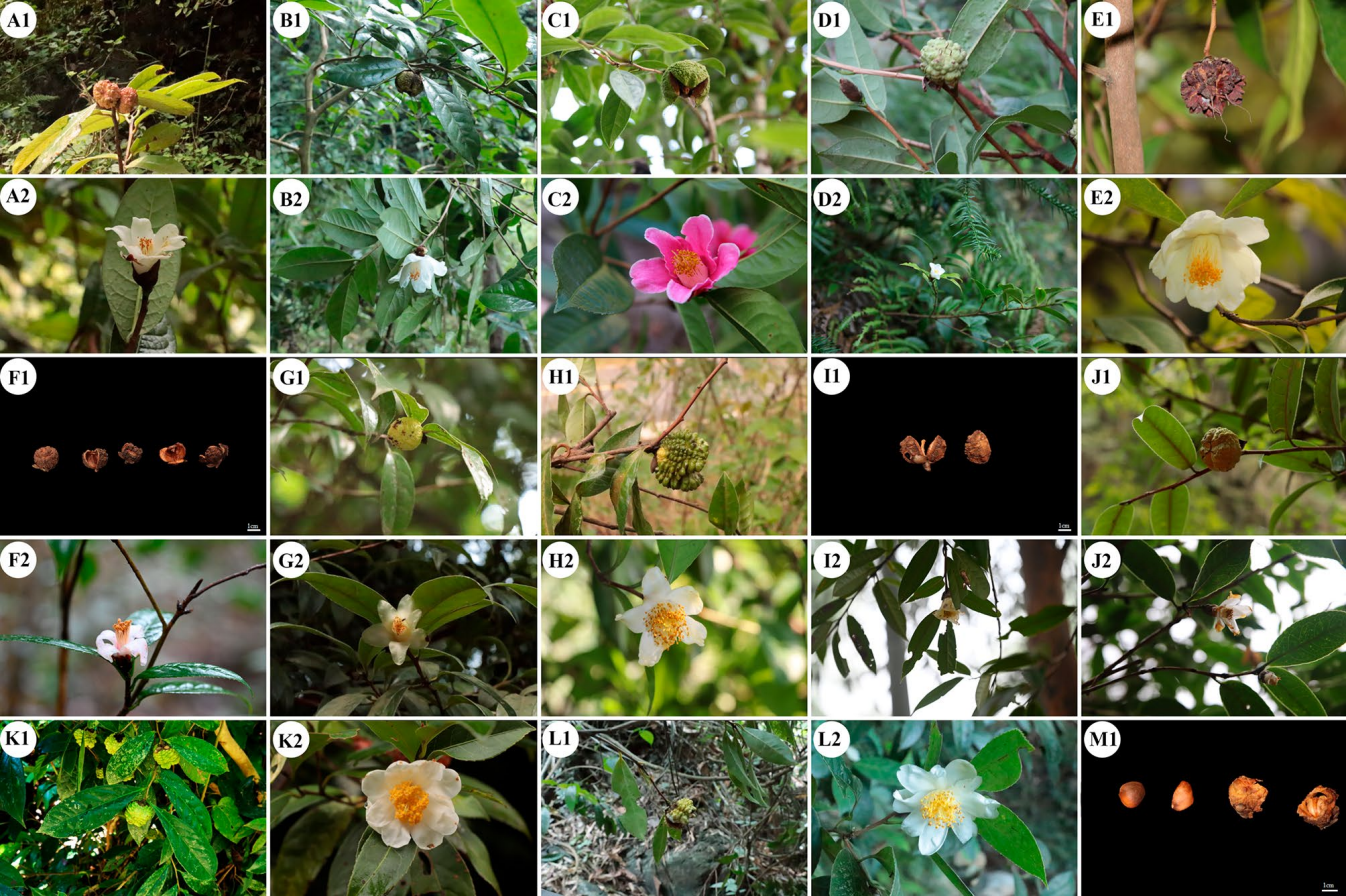
Morphological characteristics of thirteen species in sect. *Tuberculata*. (**1**: Fruit; **2**: Flower; **A**: *C. rhytidophylla*; **B**: *C. acutiperulata*; C: *C. rubituberculata*; D: *C. lipingensis*; E: *C. pyxidiacea*; F: *C. atuberculata*; G: *C. neriifolia*; H: *C. atuberculata*; I: *C. ilicifolia*; J: *C. zengii*; K: C. *anlungensis*; L: *C. leyeensis*; M: *C. rhamnoea*.)

## Materials and methods

### Plant materials and DNA extraction

In this study, thirteen sect. *Tuberculata* samples were used. The cp genome sequence of one of the samples (*C. anlungensis*) was downloaded from the NCBI database, and the other samples were collected from Guizhou and Guangxi provinces in China. Fresh, young, or old leaves in good growth condition and not contaminated by other organisms were collected from 12 plants, placed in self-sealing bags with color-changing silica gel, and stored at -80 °C until use. The specimens were arranged and preserved in the tree herbarium of the School of Forestry, Guizhou University (GZAC) (Fig. 9, Table S1). Total DNA from the 12 samples was extracted in the laboratory using the CTAB method. The purity of the DNA was determined by an Ultra-Micro spectrophotometer, and the quality of the extracted DNA was examined via 1% agarose gel electrophoresis.

### DNA sequencing, assembly and annotation

Sequencing libraries were constructed by DNA fragment end repair, ligation splicing, PCR amplification, and magnetic bead sorting to purify the ligated products. The gene library concentrations were determined using a Thermo Qubit 4.0 fluorescence quantification instrument. Libraries were sequenced using the Illumina high-throughput sequencing platform. A total of 3.7–8.37 GB of raw data were obtained (Table 1). Low-quality sequences based on quality statistics were trimmed with Trimmomatic software [46]. The clean screened cp reads were compared with published sequences from sect. *Tuberculata* in the NCBI database. A circular cp gene map was obtained by de novo splicing of the filtered data using SOAPdenovo 2 and NOVOPlasty [47, 48]. Finally, the complete cp genomes were obtained by online annotation, BLAST comparison, and manual correction using *C. rubituberculata* Chang & Yu (MZ766253) as the reference sequence. Cp genome mapping was performed using the online tool OGDRAW v1.3.1 (http://ogdraw.mpimp-golm.mp-g.de/) [49]. All annotated genomes were uploaded to NCBI, and GenBank accession numbers were obtained (Table 2).

**Table 2:** Sampling information for sect. *Tuberculata*

Number	Species	Location	Specimen number	GenBank accession number
1	*Camellia rhytidophylla* Y. K. Li &; M.Z.Yang	Kaiyang County, Guizhou, China	GZAC, LZ20220802	OQ556875
2	*Camellia acutiperulata* Chang & Ye	Longlin County, Guangxi, China	GZAC, LZ20221103	OQ556869
3	*Camellia rhytidocarpa* Chang & Liang	Longsheng County, Guangxi, China	GZAC, LZ20221106	OQ556874
4	*Camellia lipingensis* Chang	Liping County, Guizhou, China	GZAC, LZ20210830	OP004914
5	*Camellia pyxidiacea* Xu F.P. Chen & C.Y.Deng	Xingyi City, Guizhou, China	GZAC, LZ20211204	OP058659
6	*Camellia rubimuricata* Chang & Z.R.Xu	Libo County, Guizhou, China	GZAC, LZ20211213	OQ547778
7	*Camellia neriifolia* Hung T.Chang	Chishui City, Guizhou, China	GZAC, LZ20220821	OQ556872
8	*Camellia atuberculata* Chang	Chishui City, Guizhou, China	GZAC, LZ20220820	OQ556873
9	*Camellia ilicifolia* Y.K.Li	Chishui City, Guizhou, China	GZAC, LZ20221108	OQ556868
10	*Camellia zengii* Chang	Liping County, Guizhou, China	GZAC, LZ20210829	OQ556870
11	*Camellia anlungensis* Chang	Wangmo County, Guizhou, China	-	NC_050354
12	*Camellia rubituberculata* Chang & Yu	Xingren County, Guizhou, China	GZAC, LZ20210411	MZ766253
13	*Camellia leyeensis* Chang & Y.C. Zhong	Leye County, Guangxi, China	GZAC, LZ20210413	OK046127

### Repeat sequence analysis

MISA v2.1 [50] software was used to find SSRs in the organelle genomes. The minimum repeat units and number of repeats were set as follows: at least 10 for mononucleotide (mono-) repeats, at least 5 for dinucleotide (di-) repeats, at least 4 for trinucleotide (tri-) repeats, at least 3 for tetranucleotide (tetra-) repeats, at least 3 for pentanucleotide (penta-) repeats, and at least 3 for hexanucleotide (hexa-) repeats. The REPuter (https://bibiserv.cebitec.uni-bielefeld.de/reputer) [51] online tool was used to search for larger repeat sequences with a Hamming distance of 3 and a minimum repeat size of 30 bp. The four types were forward (F), reverse (R), complement (C), and palindromic (P).

### Genome structure, genome comparison and divergence hotspot identification

The boundary regions of the cp genomes of the 13 sect. *Tuberculata* plants were visualized and compared by using IRscope [52] to obtain a macroscopic view of the cp genome structure of the section. The cp genomes of 13 sect. *Tuberculata* were sequenced using MAFFT v7 [53] and imported into the mVISTA online program (https://genome.lbl.gov/vista/mvista/submit.shtml) [54] for cp genome visualization and comparison using the model Shufe-Lagan (*C. szechuanensis* C. W. Chi was used as the reference). Molecular evolutionary genetics analysis (MEGA) v11 [55] was used to analyze the codon usage distribution and GC content and for phylogenomic inference. Nucleotide diversity (Pi) analysis of the cp genome after sequence alignment was performed using DnaSP v6 [56].

### Codon usage and phylogenetic analysis

Statistical analysis of the preferential relatively synonymous codon usage (RSCU) of the cp genome codons of the 13 sect. *Tuberculata* plants was performed using CodonW 1.4.2 [57]. Phylogenetic analysis was performed using 28 cp genomes of *Camellia* species, with *Apterosperma oblata* (NC_035641) set as the outgroup. The substitution saturation indices (Iss) of the cp genomes were evaluated using DAMBE v5.3.19 [58], and the results were as follows: Iss (0.0809) < Iss.c (0.8428), *P* = 0.0000. Because the 29 cp genome sequences did not reach saturation, a phylogenetic tree was reconstructed using the maximum likelihood (ML) method in IQ-TREE v1.6.12 [59]. The optimal model (GTR + I + G) was identified using MrModeltest v2.3, and a Bayesian inference (BI) phylogenetic tree was subsequently reconstructed using MrBayes v3.2.7 [60]. The genetic distances of the thirteen sect. *Tuberculata* cp genomes after alignment were calculated by MEGA11 with 1000 bootstrap replicates.

### Electronic supplementary material

Below is the link to the electronic supplementary material.

Supplementary Material 1

Supplementary Material 2

## Data Availability

The data provided in this study were uploaded to the NCBI repository (https://www.ncbi.nlm.nih.gov/) under accession numbers NC_050354, MZ766253, OK046127, OP058659, OP004914, OQ547778, OQ556872-OQ556875, OQ556868, and OQ556869-OQ556870.
